# H3F3A (Histone 3.3) G34W Immunohistochemistry: A Reliable Marker Defining Benign and Malignant Giant Cell Tumor of Bone

**DOI:** 10.1097/PAS.0000000000000859

**Published:** 2017-05-12

**Authors:** Fernanda Amary, Fitim Berisha, Hongtao Ye, Manu Gupta, Alice Gutteridge, Daniel Baumhoer, Rebecca Gibbons, Roberto Tirabosco, Paul O’Donnell, Adrienne M. Flanagan

**Affiliations:** * Royal National Orthopaedic Hospital NHS Trust, Stanmore, Greater London; † Cancer Institute, University College London, London, UK; ‡ Basel Bone Tumour Reference Centre (BBTRC), University Hospital Basel and University of Basel, Basel, Switzerland

**Keywords:** bone tumor, giant cell tumor, osteosarcoma, *H3F3A*, G34W

## Abstract

Giant cell tumor of bone (GCTB) is a locally aggressive subarticular tumor. Having recently reported that *H3.3* G34W mutations are characteristic of this tumor type, we have now investigated the sensitivity and specificity of the anti-histone H3.3 G34W rabbit monoclonal antibody in a wide variety of tumors including histologic mimics of GCTB to assess its value as a diagnostic marker. We also determined the incidence of *H3.3* G34 mutations in primary malignant bone tumors as assessed by genotype and H3.3 G34W immunostaining. A total of 3163 tumors were tested. Totally, 213/235 GCTB (90.6%) showed nuclear H3.3 p.G34W immunoreactivity. This was not the case for the rare variants, p.G34L, M, and V, which occurred most commonly in the small bones of the hands, patella, and the axial skeleton. If these sites were excluded from the analysis, H3.3 G34W expression was found in 97.8% of GCTB. Malignant bone tumors initially classified as osteosarcomas were the only other lesions (n=11) that showed G34W expression. Notably an additional 2 previously reported osteosarcomas with a p.G34R mutation were not immunoreactive for the antibody. A total of 11/13 of these malignant H3.3-mutant tumors exhibited an osteoclast-rich component: when imaging was available all but one presented at a subarticular site. We propose that subarticular primary malignant bone sarcoma with *H3.3* mutations represent true malignant GCTB, even in the absence of a benign GCTB component.

Giant cell tumor of bone (GCTB) is a locally aggressive primary bone tumor that is characteristically sited in the subarticular (epiphyseal/epimetaphyseal) location. The distal femur and proximal tibia are the most common sites but the distal radius and proximal humerus are not uncommonly affected.^1^ In the axial skeleton, the proximal sacrum is the most common location but GCTB can occur in almost any bone, including the small bones of the hands and feet, and sesamoid bones such as the patella.^2^–^4^ Patients are most frequently affected in their third and fourth decades and there is a slight female predominance (1.5F:1M). Reports of GCTB in the immature skeleton are extremely rare.^5^ GCTB is one of the few “benign” tumors that occasionally metastasizes to the lungs.^6^

In the classical clinical and radiologic settings, GCTB does not pose a major diagnostic challenge. However, due to the morphologic intratumoral heterogeneity, including secondary aneurysmal bone cyst (ABC) change, extensive infarction with associated fibroblastic overgrowth and high mitotic activity, it may be difficult to reach a definitive diagnosis particularly on small biopsies.^1^ The differential diagnoses include the numerous osteoclast-rich lesions of bone: chondroblastoma, nonossifying fibroma (NOF), primary ABC, giant cell lesions of the small bones and jaw (giant cell granulomas), brown tumor of hyperparathyroidism and tenosynovial giant cell tumor invading bone. The greatest of these challenges involves distinguishing conventional GCTB from malignancy in a GCTB, and from an osteoclast-rich osteosarcoma, particularly on a biopsy.^7^–^9^ Furthermore, there is no consensus on the best treatment for malignancy in GCTB.^6^,^8^–^11^

Malignancy in a GCTB is characterized by areas of high-grade sarcoma juxtaposed to a conventional GCTB.^1^ The malignant component does not have a specific phenotype: it may be osteoclast-rich and tumor osteoid may be evident. Most cases arise de novo (primary malignancy in GCTB) when the atypical features are identified at presentation. Secondary malignancy in GCTB presents on tumor recurrence of a previously conventional GCTB, which has usually been treated with irradiation or surgery. Malignancy in GCTB is uncommon and reported to occur as rarely as <2% to up to 9% of GCTB.^7^–^9^,^12^

We have recently reported the presence of a histone *H3F3A* (H3.3) gene mutation involving a substitution in glycine 34 in 96% of GCTB, the vast majority of which are represented by p.Gly34Trp (p.G34W), although small numbers of G34L, V, and M variants have been detected.^13^,^14^ We also published that chondroblastoma harbors a H3.3 p.Lys36Met (p.K36M) mutation, generally in the *H3F3B* gene, although occasionally in H3.3A, in over 90% of cases. These *H3.3* mutations are mutually exclusive and appear to be rare events outside these 2 tumor types.^15^,^16^ Importantly, the neoplastic element of the tumor is represented by the stromal mononuclear cells and not the osteoclasts.^13^

We have previously reported *H3.3* p.G34 mutations in 2% of osteosarcoma (2/103): both had a G34R substitution^13^ and others have reported another 2 cases of malignant osteoclast-rich tumors with a G34W substitution.^14^ In addition, *H3.3* p.G34W has been implicated in a new mosaic disorder characterized by pheochromocytomas, paragangliomas, and GCTB.^17^ To our knowledge *H3.3* p.G34 mutations have not been reported in malignancy in GCTB although the *H3.3* pG34 mutations reported in conventional GCTB have been reported in “symplastic/pseudoanaplastic GCTB of bone”: this is a rare entity describing the presence of scattered atypia and “smudged” cells in an otherwise typical GCTB.^18^

We have already demonstrated the clinical benefit of a rabbit monoclonal histone H3F3 K36M antibody when diagnosing chondroblastoma.^19^ Having an antibody for detection of H3.3 p.G34W would also be valuable as a diagnostic tool.

In this study we investigated sensitivity and specificity of the anti-histone H3.3 G34W Rabbit monoclonal antibody (clone RM263; RevMAb Biosciences USA, San Francisco, CA) in a wide variety of tumors, including histologic mimics of GCTB to assess its value as a diagnostic marker, with a particular attention to biopsy material. A major focus of the study involved genotyping and immunostaining a series of osteosarcomas to determine the incidence of *H3.3* G34 mutations in these primary malignant bone tumors.

## MATERIALS AND METHODS

Cases of interest were identified by searching the histopathology archives at the Royal National Orthopaedic Hospital, UK and Basel Bone Tumour Reference Centre, Switzerland. Samples were obtained from the Stanmore Musculoskeletal Biobank, a satellite of the UCL/UCLH Biobank (HTA License number 12055), which was approved by the National Research Ethics Committee (reference 15/YH/0311). This specific study was approved by the NREC-approved UCL/UCLH Biobank Ethical Review Committee (reference EC17.14), and from the Ethikkommission beider Basel (reference 274/12). The tumors were classified according to the 2013 World Health Organization classification of soft tissue and bone tumors.^20^

All samples were diagnosed by specialist bone tumor pathologists at RNOH (A.M.F., F.A., and R.T.), and at BBTRC (D.B.) according to the current World Health Organization classification. Individual cases were correlated with clinical and radiologic information where available. Tissue sample types and numbers are listed below. In cases where the histology and genotyping did not match, the morphology was reevaluated independently by all members of the RNOH pathology team and a consensus reached.

All samples were fixed in 10% formal saline and processed in paraffin wax after which the tissue sections were stained with hematoxylin and eosin. The standard operating procedure for decalcification at RNOH involves the use of EDTA for at least a part of the specimen, even if heavily calcified. To accelerate the decalcification process at least part of heavily calcified samples are decalcified in nitric acid (5%).

Either full tissue sections or sections from previously constructed tissue microarrays were cut for immunohistochemistry, as previously described.^21^ In brief, tissue microarrays in formalin-fixed paraffin-embedded blocks were built with a manual arrayer (Beecher Instruments, Sun Prairie, WI). Duplicate 0.6 to 1.0 mm diameter cores were taken from each case. All biopsies were tested in full sections.

The anti-histone H3.3 G34W Rabbit monoclonal antibody (clone RM263; RevMAb Biosciences USA) (diluted 1:1500), and the rabbit monoclonal histone H3F3 K36M antibody (clone RM193; RevMAb Biosciences USA) (diluted 1:400) were both kindly provided as a gift by RevMAb Biosciences USA. Immunohistochemistry was performed with the Leica Bond 3 fully automated immunohistochemistry stainer (Leica Microsystems, Milton Keynes, UK). Leica epitope retrieval 2 solution was used for 20 minutes. Immunohistochemical reactions were characterized as positive when there was unequivocal strong crisp nuclear expression and negative when expression was not detected. Control material was a GCTB and a chondroblastoma as appropriate in which the relevant mutation had been demonstrated by DNA sequencing and which revealed strong and crisp nuclear expression.

Genotyping of the *H3.3* G34W, and *H3.3* K36M mutations was undertaken using a variety of techniques including either digital polymerase chain reaction, targeted next generation sequencing with the Ion Personal Genome Machine, whole genome sequencing, and whole exome sequencing.^13^,^14^ *USP6* gene rearrangement analysis was performed using interphase fluorescence in situ hybridization analysis (ZytoLight SPEC USP6 Dual Color Break Apart Probe; Zytovision, Bremerhaven, Germany) as previously reported.^22^,^23^

A total of 3163 cases were tested for the expression of *H3.3* G34W and included 235 GCTB from common anatomic sites (femur, tibia, radius, ulna, fibula, humerus, pelvis, and sacrum: 206 tumors), as well as less frequent sites (29 tumors) including vertebrae, clivus, short bones of the hands and feet, and patella. Included in these 235 tumors was the validation group of 138 tumor samples (see below). The age at presentation of the GCTB cohort ranged from 13 to 76 years old (mean: 34.7 y): these occurred in 128 men and 107 women (1.2M:1F ratio).

A total of 750 mimics of GCTB were also tested, including primary ABC (n=55), chondroblastoma (all H3.3 K36M immunoreactive) (n=58), giant cell granulomas of the jaw (n=99), cherubism with a *SH3BP2* alteration (n=3), chondromyxoid fibromas (n=45), NOF (n=28), osteoblastomas (n=27), osteosarcomas (n=385), tenosynovial GCT (n=46), foreign body-type giant cell reaction (n=1), and brown tumors of hyperparathyroidism (n=3). An additional group of 2,178 tumors tested for H3.3 G34W occurring in bone and soft tissue, included angiosarcomas (n=31), carcinomas (breast, prostate, renal, bowel, and squamous) (n=519), clear cell sarcomas (n=16), chordomas (n=494), conventional cartilaginous tumors (enchondromas and chondrosarcomas) (n=86), extraskeletal myxoid chondrosarcomas (n=30), fibrous dysplasia (n=56), gastrointestinal stromal tumors (n=52), Langerhans cell histiocytosis (n=7), leiomyosarcomas (n=83), spindle cell lipomas (n=5), dedifferentiated liposarcomas (n=13), pleomorphic liposarcomas (n=24), mesenchymal chondrosarcomas (n=7), melanomas (n=26), myxofibrosarcomas (n=162), malignant peripheral nerve sheath tumor (n=285), neurofibromas (n=57), phosphaturic mesenchymal tumors (n=9), plasma cell tumors (n=8), undifferentiated pleomorphic sarcomas (n=44), solitary fibrous tumors (n=42), soft tissue chondromas (n=18), soft tissue giant cell tumors (n=2), soft tissue osteosarcomas (n=10), and synovial sarcomas (n=92).

### Validation of the H3.3 G34W Antibody

Genotyping identified the *H3.3* p.G34W mutation in 138 neoplasms with typical histologic features of GCTB on a resection or curettage specimens. All except one of these 138 tumors was immunoreactive for this protein and none expressed the H3.3 K36M mutant protein characteristic of chondroblastoma, nor exhibited an *USP6* rearrangement, characteristic of primary ABC. Furthermore, 50 cases of GCTB which exhibited strong nuclear expression for H3.3 G34W failed to reveal a K36M substitution by genotyping.

### Imaging

Imaging was reviewed when there was a discrepancy between the diagnosis of a GCTB and the absence of immunoreactivity for H3.3 G34W, and in cases diagnosed before the age of 18 years.

## RESULTS

**FIGURE 1:**
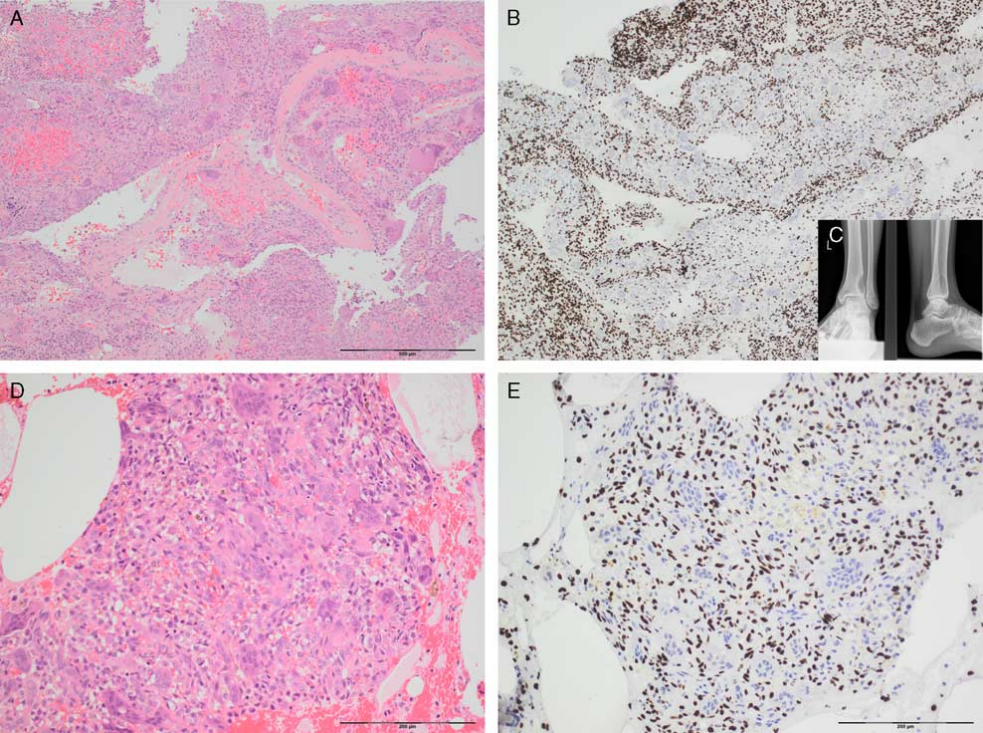
Photomicrographs (A and B) and radiographs (C) of a GCTB with extensive secondary aneurysmal bone cyst change, in areas indistinguishable from a primary aneurysmal bone cyst. H3.3 G34W is diffusely expressed by the stromal cells. D and E represent photomicrographs, of a different case (biopsy sample) showing a small isolated fragment of an osteoclast-rich lesion within blood clot. Strong H3.3 G34W (E) nuclei expression confirms the suspicion of a GCTB. The osteoclasts are negative for H3.3 G34W expression.

Of the entire set of 235 GCTB analyzed 213 (90.6%) revealed strong nuclear expression for H3.3 G34W (Fig. 1). Of the remaining 22 negative samples, 6 harbored another G34 substitution (2x p.G34V, 3x p.G34L, and 1x p.G34M): genotyping gave a noninformative result in 3 cases, and 1 case with a DNA sequenced-proven G34W mutation was negative on immunohistochemistry (Table 1). The outstanding 12 cases (5.1% of the total group of 235) were found to be wild-type for *H3F3A* G34 and negative for both the characteristic *USP6* gene rearrangement found in ABC and H3.3 K36M substitution seen in chondroblastoma. Hence, when the noninformative cases are excluded, 94.8% of GCTB harbor a H3.3 G34 mutation.

**TABLE 1:**
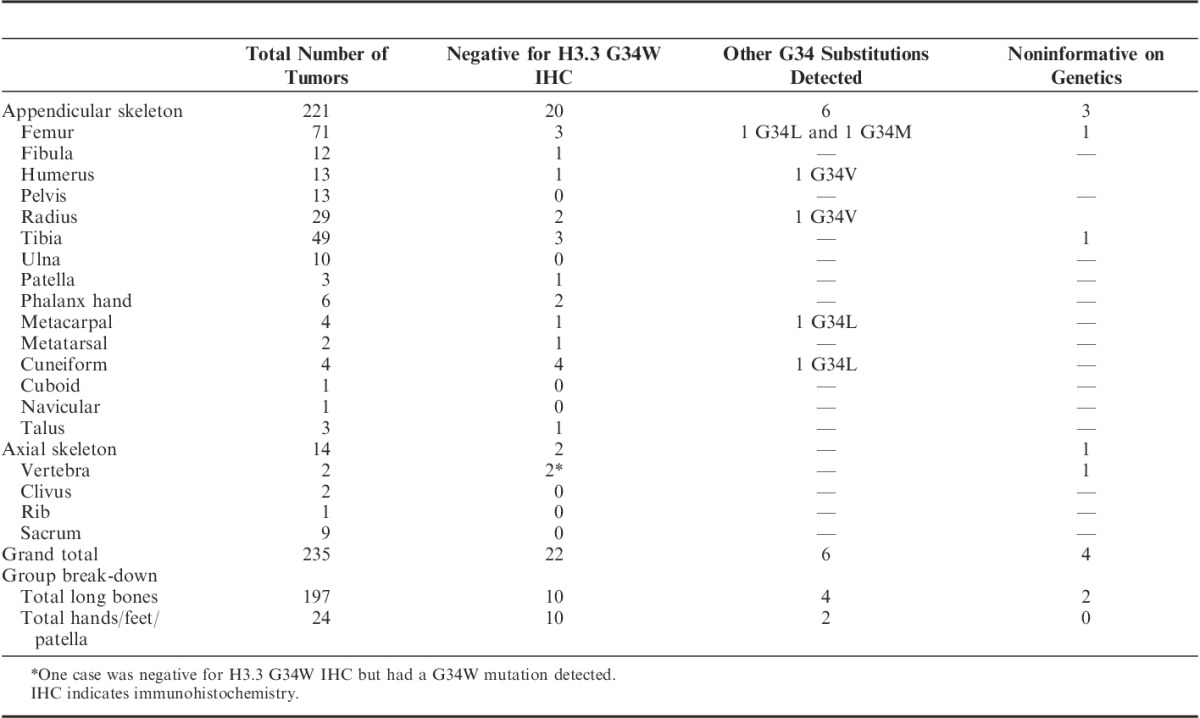
Giant Cell Tumors of Bone

We next classified the 235 GCTB on the basis of anatomic site (Table 1): 184 tumors occurred in long appendicular bones (femur, tibia, radius, ulna, humerus, and fibula) of which 174 (94.6%) expressed H3.3 G34W. Of the 10 negative cases, 4 revealed a different G34 substitution (x2 p.G34V, x1 p.G34L and x1 p.G34M). When excluding the 2 cases that were noninformative on genotyping, only 2.2% (4 cases) of the 235 GCTB of the long appendicular bones were wild-type for G34. In addition, all GCTB from the sacrum (n=9), and the pelvis (ilium, ischium) (n=13) were immunoreactive for G34W. In contrast 60% (n=3/5) and 58.3% (n=14/24) of GCTB of the axial skeleton (excluding sacrum and pelvis), and the small bones of the hands and feet and patella, respectively were found to be wild-type for H3.3 G34 (Table 1).

The *H3.3* G34W mutant tumors exhibited strong and diffuse nuclear immunoreactivity in the vast majority of cases (Fig. 1). In 3 cases, the immunostaining was weaker than generally observed, and was present in a patchy distribution, largely seen at the periphery of the tissue section, but still was unequivocally positive. On investigation, these 3 cases were found to have been subjected to harsh acid-based decalcification, and notably they were also noninformative on genotyping.

### Mimics of GCTB

Study of the mimics of GCTB revealed 3 tumors that expressed *H3.3* G34W. mutant tumors On review the diagnoses were altered to GCTB. One was originally interpreted as an unusual osteoblastoma of the rib and 2 cases as ABC. The latter were sited in the distal femur and exhibited secondary ABC-like change, and a *USP6* translocation was not detected by fluorescence in situ hybridization.

### Malignant Bone Tumors With *H3.3* G34 Mutations

**FIGURE 2:**
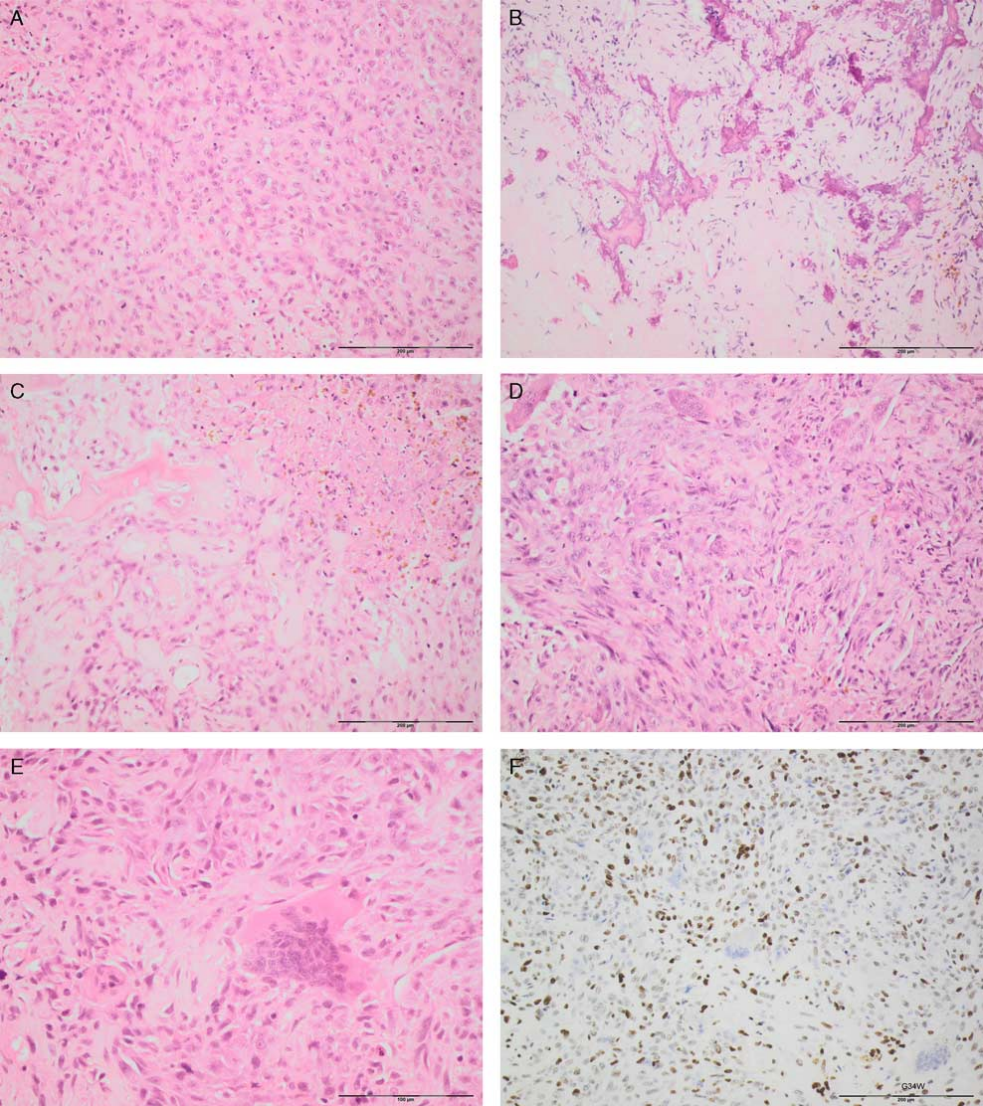
Photomicrographs of a malignant GCTB (M-07: initially classified as osteoclast-rich osteosarcoma) of the distal tibia showing morphologic variation including areas composed of epithelioid cells arranged in cords (A–C), osteoid deposition (B and C), tumor necrosis (C), and spindle cells with moderate nuclear atypia and numerous interspersed osteoclasts (D–F). Diffuse expression of H3.3 G34W is seen in the stromal cells (F).

**FIGURE 3:**
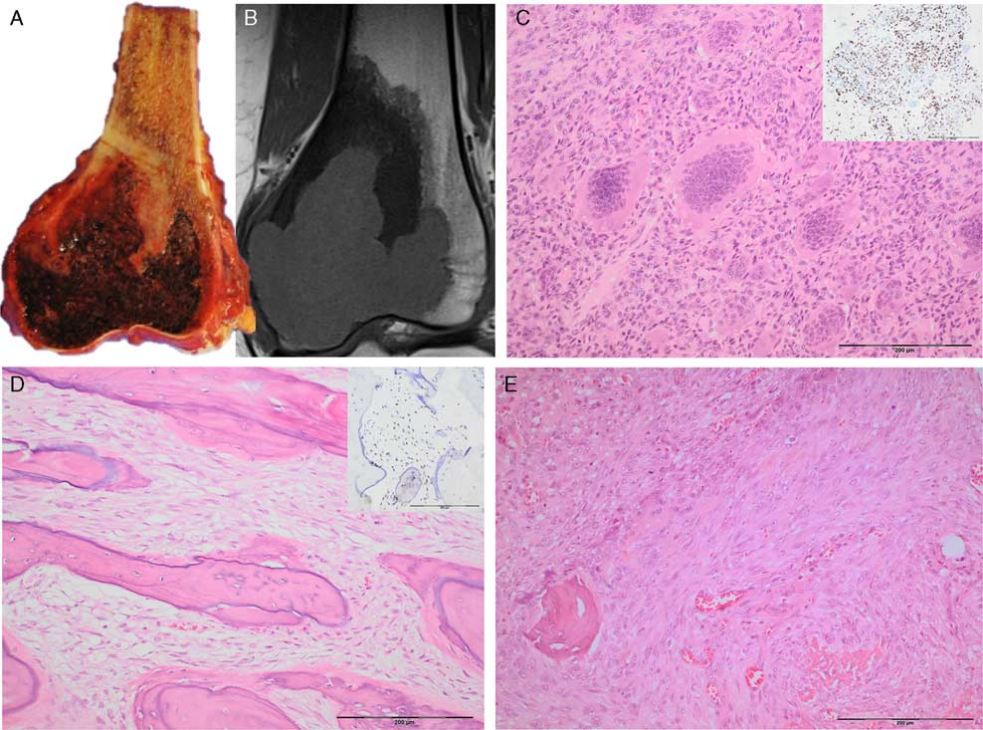
A and B, Resection specimen and corresponding coronal T1 MRI scan showing a lytic hemorrhagic lesion in the subarticular area of the distal femur with a surrounding low signal (sclerotic) component at the proximal aspect of the lesion. Photomicrographs of the subarticular component shows a conventional GCTB with diffuse H3.3 G34W expression (inset) (C). The proximal component comprises a bone-forming tumor with a spindle cell stroma showing a permeative growth pattern (D and E) (H3.3 G34W expression shown on inset D) case M10.

Of the 2928 non-GCTB tumors the only subtype that revealed H3.3 G34W immunoreactivity was osteosarcoma. Of the 385 osteosarcomas of the skeleton 11 (2.85%) revealed G34W immunoreactivity (Fig. 2 and Table 2). Of these one fulfilled the WHO criteria of primary malignant GCTB with the malignant component being a low-grade central OS which tested negative for *MDM2* gene amplification (Fig. 3). Nine of the remaining 10 OS had an osteoclast-rich component, and 1 was a chondroblastic osteosarcoma. All 9 cases for which macroscopic and/or radiologic information was available, were sited in the subarticular area. Of the 385 cases, 237 were also genotyped (103 previously performed^13^,^14^) and the absence of G34W substitution supported the lack of immunoreactivity in the majority of osteosarcomas. The genotyping also revealed a *H3.3* G34 alteration in 2 cases (both with H3.3 G34R mutations—one in *H3F3A* and 1 in *H3F3B*, both reported previously^13^) but no other G34 substitutions. One of these was a subarticular osteoclast-rich osteosarcoma. Combining the results, 13/385 (3.37%) primary malignant bone tumors classified as either osteosarcoma or malignant GCTB harbored a p.G34 alteration. The p.G34W mutation was 4 times more common than the other G34 substitutions. None of the primary tumors had been treated with radiotherapy. Table 2 provides details of these 13 cases.

**TABLE 2:**
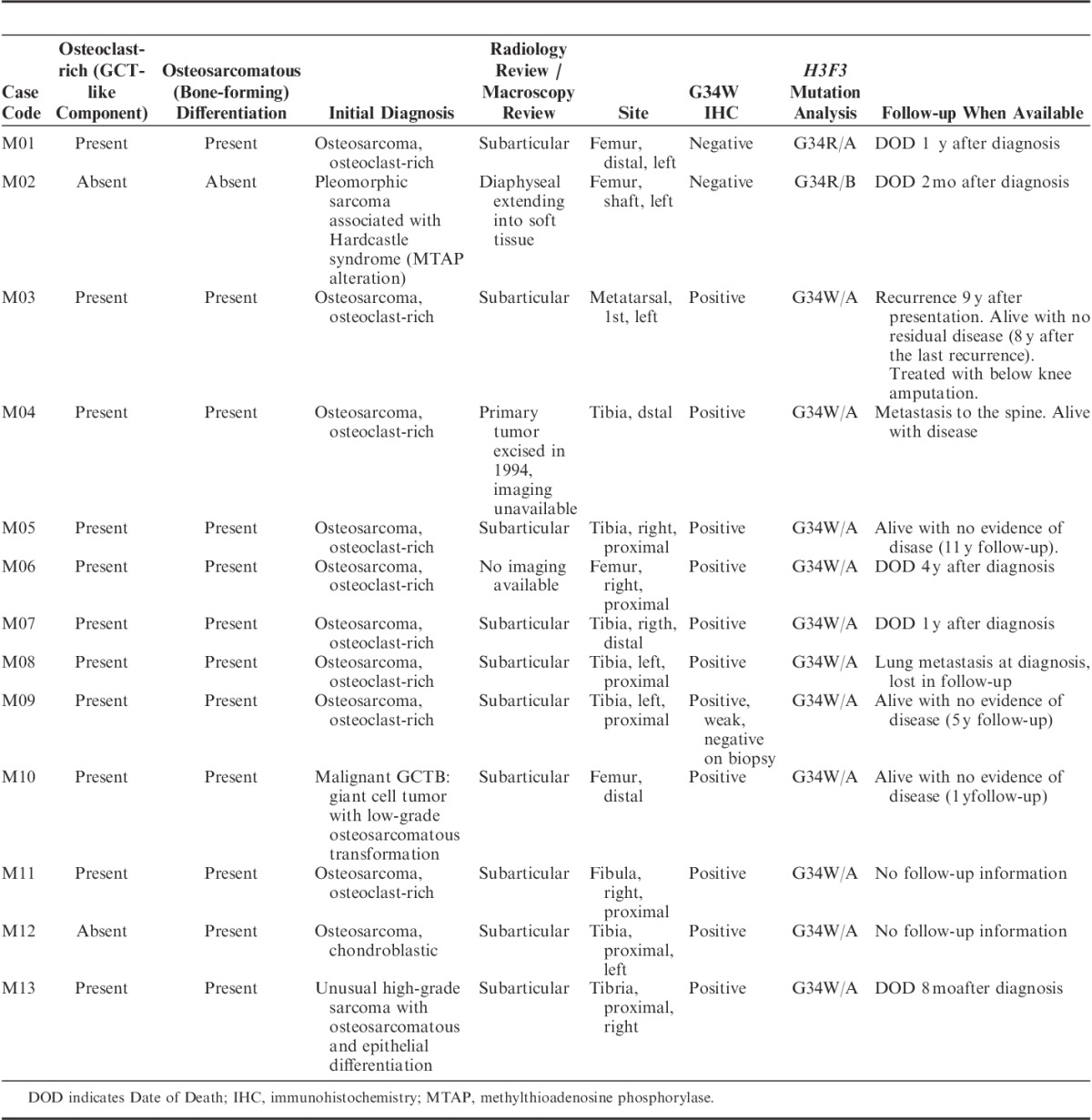
Malignant Bone Tumors With G34 Alterations

The average age at presentation for malignant tumors with H3.3 mutations was 34 (range: 19 to 64): 4M:9F.

### H3.3 G34W Immunohistochemistry on Biopsy Material

To determine the value of the H3.3 G34W immunohistochemistry for diagnosing GCTB on biopsy specimens, we analyzed 94 consecutive osteoclast-rich biopsies samples over an 18-month period. The resection/curettage specimens related to these biopsies were also tested for G34W immunoreactivity (Table 3). A definitive diagnosis of GCTB on biopsy could have been increased by 5 cases with the aid of immunohistochemistry. Of the 59 cases where the diagnosis of GCTB was provided on resection/curettage, H3.3 G34W was expressed in 57 (96.6%).

**TABLE 3:**
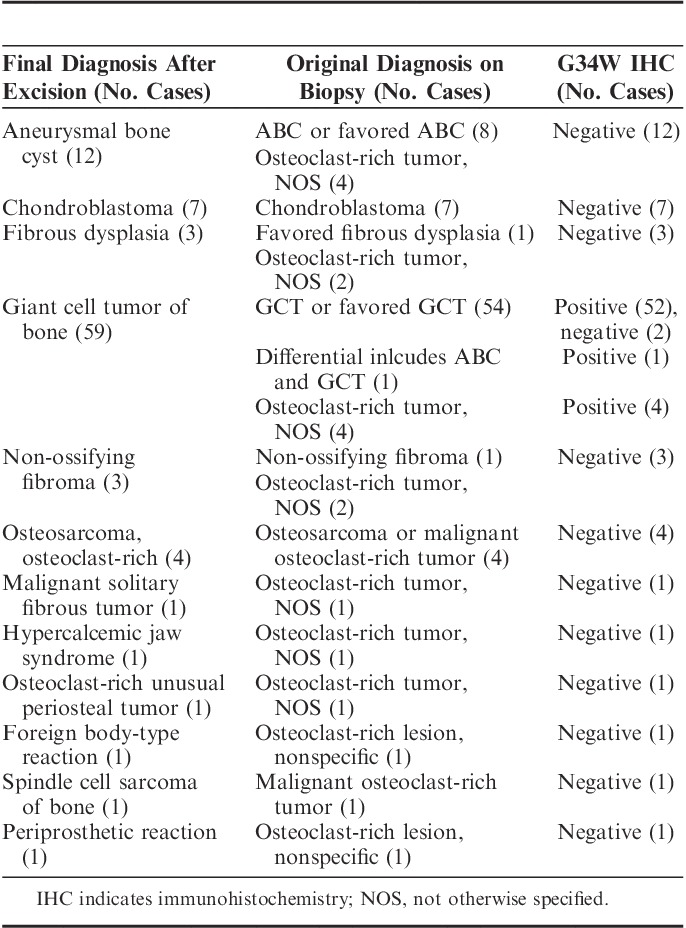
Giant Cell-rich Lesions of Bone: Needle Biopsies (18 mo)

### Imaging

Five cases in our cohort presented in patients 15 years old or younger: the youngest presented at 13. The growth plates were open in 2 patients: one 14-year-old girl (tumor in the distal radius, adjacent growth plates open) and the other a 15-year-old boy (right cuboid tumor, distal tibial, and fibular growth plates open). The other patients had closed growth plates. The malignant tumors harboring a *H3.3* mutation were usually accompanied by aggressive radiologic features which were not compatible with a diagnosis of conventional GCTB.

## DISCUSSION

This study demonstrates that the anti-histone H3.3 G34W Rabbit monoclonal antibody (clone RM263) is highly specific and sensitive for tumors harboring this mutation and is a powerful marker for GCTB, significantly improving the diagnostic accuracy in biopsy specimens. H3.3 G34W expression is seen exclusively in the mononuclear cell population confirming our previous finding by genotyping that the neoplastic cell population is represented by the mononuclear cells and not the osteoclast-like giant cells.

The sensitivity of the antibody for the mutant protein was verified by testing 138 GCTB previously genotyped for the *H3.3* G34W substitution: only 1 case (1/138) failed to demonstrate immunoreactivity which is likely to be explained by tissue fixation and decalcification, highlighting the importance of establishing procedures for tissue handling. The specificity of the antibody for the mutation was demonstrated by absence of H3.3 G34W expression in the cases proven to harbor a *H3.3* G34L, M, and V mutations in GCTB, and *H3.3* G34R in 2 primary malignant bone tumors. Furthermore, >2500 tumors other than GCTB were not found to be immunoreactive for this antibody including several subtypes previously shown to be wild-type for *H3.3* G34 by next generation sequencing (ie, chondroblastomas, chondrosarcoma, osteoblastomas, NOF ABC, and chordomas).^14^ Having established the quality of this histone G34W antibody, we have demonstrated that it is immunoreactive for 90.6% of 235 GCTB, confirming the sensitivity we previously reported by genotyping.^14^ It is noteworthy that the rare *H3.3* variants including G34L, M, and V occurred most commonly in the small bones of the hands and feet, patella and the axial skeleton, and if these anatomic sites were excluded from the analysis, the G34W mutation is found in 97.8% of GCT. The absence of H3.3 G34 mutation in GCTB may be explained by a different genetic alteration. Alternatively, they may represent solid variants of an ABC without a *USP6* gene rearrangement.

GCTB have rarely been reported in the immature skeleton: of the 235 cases in our study, 13 years of age was the earliest age at presentation and there was a total of 13 (5.5%) patients presenting at 18 years of age or younger. This number is slightly lower than that reported recently by Al-Ibraheemi et al.^5^ However, the age of presentation seems less important than skeletal maturity and in only 2 individuals in our cohort were the physes still open at presentation. GCTB at a metaphyseal site in patients with open physes is well-documented,^24^ and is exemplified in 1 case.

In addition to the 95% of conventional GCTB in which a *H3.3* G34 mutation was detected we also identified 13 primary malignant bone tumors harboring a G34 mutation. Eleven of those harbored a G34W mutation, and 2 had a G34R alteration (Table 2). The average age at presentation for benign and malignant tumors with a *H3.3* G34 mutation was not dissimilar and the distinguishing feature between these tumor types was that the immunoreactive mononuclear tumor cells in the malignant tumors displayed significant cytologic atypia, not seen in conventional GCTB. Using the criteria of the current WHO classification, 11 of these tumors would be classified as osteosarcoma, 1 as a pleomorphic undifferentiated sarcoma of bone, and 1 as malignancy in a GCTB. However, it was remarkable that osteoclast-rich areas were observed in all but 1 of these 13 cases and that where imaging and or macroscopic images (9/11) were available for review, all were sited in the subarticular region. The one case in which an osteoclast-rich component was not detected despite extensive sampling was sited in the diaphyseal region and presented in a patient with Hardcastle syndrome, characterized by diaphyseal medullary stenosis (sclerosis) with bone malignancy (malignant fibrous histiocytoma), a germline disorder revealing structural alterations in the gene encoding methylthioadenosine phosphorylase.^25^

There was only one tumor which revealed histologic features of a typical GCTB in addition to a frankly malignant element in the tumor. In both components, the mononuclear cell population was immunoreactive for the anti-histone antibody. In the other 11 tumors, the stromal component revealed unequivocal malignant morphology in the mononuclear cells throughout the lesion. The single case that fulfilled the WHO criteria for malignancy in GCTB was also noteworthy on account of the malignant component displaying a low-grade fibroblastic bone-forming neoplasm reminiscent of a low-grade central osteosarcoma, a histologic variant in malignant GCTB not reported previously. The finding of the *H3.3* mutation in the stromal component of both the benign and malignant components argues for transformation of the tumor presumably brought about by acquisition of additional genetic alterations, as yet undefined; this also supports the concept that this the *H3.3* mutation is a driver mutation.

Although the numbers of primary malignant bone tumors with *H3.3* mutations in our study is small, there is clear variation in the clinical behavior within the group with the majority behaving as high-grade bone tumors. The disease behaved aggressively in 6/10 “malignant GCTB” cases following presentation but in 3 cases the disease has not progressed and in 1 case the patient has lived with metastatic disease to the spine for more than 10 years. The clinical course of the disease was not known in 2 patients as they were lost to follow-up.

Our findings beg the questions as how best to classify primary malignant bone tumors with H3.3 G34 mutations, and most importantly how best to treat the disease. To address the first question we propose that the lack of a benign GCTB component in these tumors could be considered analogous to a dedifferentiated chondrosarcoma in which the cartilaginous component is not identified, a finding that is explained by over-growth by the more aggressive elements of the tumor. Hence, we propose that a primary malignant bone tumor when presenting in a subarticular site with a *H3.3* G34 mutation is classified as malignancy in GCTB.

To address the issue of how best to treat malignant tumors with *H3.3* G34 mutations, we propose that management is based on the histologic grade of the tumor and that high grade lesions are treated with standard of care neoadjuvant chemotherapy given for osteosarcoma. This would be in line with how any high-grade primary bone sarcoma is treated, even those with very little or no osteoid deposition such as telangiectatic osteosarcoma and the rare primary fibrosarcoma of bone. If this were done, gathering data from multiple centers could potentially identify in a relatively short period how this genetically characterized tumor responds to chemotherapy. Furthermore, with more widespread epi/genomic analysis it is likely that additional epi/genetic events that account for their malignant transformation will be identified and used as part of the diagnostic and prognostic armory.

In summary, our findings provide strong evidence that the anti-histone G34W antibody is a valuable tool for supporting a diagnosis of GCTB and distinguishing GCTB from its mimics. The limitation of this antibody is that a small number of GCTB harbor a G34 mutation, other than G34W that cannot be detected. Importantly, the same G34 mutations occur in a small number of primary malignant bone tumors. Finally, the frequent association of the *H3.3* G34 mutation with the presence of osteoclasts implies that this mutant protein plays an important role in bone biology. Understanding how the epi/genetic alterations in H3.3 are involved in the regulation of osteoclast recruitment would shed light on the bone cell biology in health and disease.
